# Influence of immigration on prematurity in the context of a free healthcare system with universal coverage.

**DOI:** 10.1038/srep10586

**Published:** 2015-05-22

**Authors:** Ernesto Cortés, María Mercedes Rizo-Baeza, Antonio Palazón-Bru, María José Aguilar-Cordero, Vicente Francisco Gil-Guillén

**Affiliations:** 1 Department of Pharmacology, Paediatrics and Organic Chemistry, Miguel Hernández University, San Juan de Alicante, Spain; 2 Department of Nursing, University of Alicante, San Vicente del Raspeig, Spain; 3 Department of Clinical Medicine, Miguel Hernández University, San Juan de Alicante, Spain; 4 Department of Nursing, University of Granada, Granada, Spain

## Abstract

We assessed the risk of preterm birth according to the mother’s place of origin in the context of a free and universal healthcare system. We analysed 75,292 newborn infants born between 2008-2011 in Alicante (Spain). The outcomes were: 1) very preterm (gestational age ≤32 weeks) and 2) moderate-to-late preterm (gestational age 33-37 weeks). Other variables: infant’s gender, maternal age and origin. We estimated adjusted odds ratios to analyse the relationship between the outcomes and the other variables. The distribution of the gestational age groups in our sample was: very preterm, 812; moderate-to-late preterm, 5,295; full-term, 69,997. There were no statistically significant differences between the mother’s place of origin and the outcomes in this free universal healthcare system, which is experiencing the recent phenomenon of immigration. This equality should be maintained throughout the time the immigrants remain in the country.

Preterm birth is a syndrome with multiple causes^1^, one of which is a low socio-economic level^2^, often associated with being an immigrant^3^. Several studies have been undertaken in different countries in a setting of universal free healthcare, mainly in Europe and Canada, assessing the influence of immigration on preterm births, considering gestational age or low birth weight. Most of these studies found a greater ratio of preterm births in immigrant mothers^3^,^4^,^5^,^6^,^7^,^8^,^9^,^10^,^11^,^12^,^13^,^14^, though there was a lack of concordance among the studies undertaken in the Mediterranean area^9^,^10^. The association between immigration and prematurity has also been studied in countries with other types of healthcare system, such as the United States, where differences were found in comparison with native (non-immigrant) mothers^14^.

Alicante is a Mediterranean province in Spain which, like the rest of Spain, has seen the arrival of many immigrants over the last 10 years; three in every four immigrants arrived between 1997 and 2007^15^. Furthermore, in Alicante there has been a high percentage (Total, 24.4%; Rest of Europe, 8.5%; Ibero-America, 7.8%; Africa, 6.8%; and Asia, 1.3%) of births to female immigrants in recent years^16^. In Spain the healthcare system is free with universal coverage for pregnant women and infants, even when their immigration status is irregular.

The possible repercussions of immigration on preterm births have been widely studied, but this relation still remains unclear in Mediterranean countries, as whilst an association was found in France, no association was seen in Italy^9^,^10^. To shed more light on this situation, we undertook a study in the province of Alicante (Mediterranean area) to determine the risk of prematurity according to the mother’s place of origin. We hypothesized that the mother’s place of origin was not a factor predisposing to premature delivery given equality of healthcare conditions for the entire population.

## Methods

### Study design and participants

This case-control study was conducted in the province of Alicante, where there is a high percentage of births to immigrant mothers (25%) from different countries. We analysed a sample of infants born between 2008 and 2011 who were registered in the neonatal screening program in Alicante, which covers over 99% of the population. This screening program covers all infants, independently of the type of hospital where they are born (public, fully private, or subsidised private).The sample consisted of all infants with a gestational age (GA) <37 weeks (cases) and a random sample of full-term births (controls). This latter sample was obtained from stratified sampling to randomly select 25 full-term births in each month of the four-year study period. Still births are not referred for neonatal screening and were not therefore included in this study.

### Variables and measures

Gestational age was measured in weeks for each infant using ultrasound evaluation^17^, and this value was used to define the main study parameters: i) very preterm (VPT) (GA ≤ 32 weeks) and ii) moderate-to late-preterm (MLPT) (GA <37 weeks). These GA intervals were selected in accordance with the Standards Committee of the Spanish Society of Neonatology^18^. We also recorded data on the infant’s gender, mother’s age (in years) and mother’s place of origin. We calculated the square of the mother’s age, as the relationship of maternal age to preterm birth is known to be U-shaped^19^. Since there was such a wide variety of nationalities, these were grouped according to the census conducted by the Department of Health of the Valencian Community^16^, obtaining the following figures for the mother’s place of origin: Spain, 75.8%; Africa, 6.8%; Ibero-America, 7.8%; Asia, 1.3%; Rest of Europe, 8.5%. More details about the countries involved in each group can be seen in the Supplementary Note.

### Sample size

The total sample size was 4,324 infants, of whom 2,661 were MLPT and 588 were VPT. Based on these figures, sample size calculations for each parameter are listed individually: i) VPT: using a 95% confidence interval, the relationship between cases and controls in the total sample, and an exposure ratio of 23% in cases and 11.4% in controls to estimate the OR, we obtained a statistical power close to 100%; and ii) MLPT: using a 95% confidence level, the relationship between cases and controls in the total sample, and an exposure ratio of 77% in cases and 82.1% in controls, we obtained a statistical power greater than 95%. These exposure percentages were obtained from data on 1,000 children selected at random from the total sample, where being from Africa was considered the attribute (exposure) and MLPT and VPT the outcome (case).

### Statistical analysis

Absolute and relative frequencies were used to describe the qualitative variables, and means and standard deviations to describe the quantitative variables. Multivariate logistic regression models adjusted for oversampling were used to estimate adjusted odds ratios (ORs), with the aim of analysing the relationship between the main study variables (MLPT and VPT) and the other variables. The ORs were adjusted for infant gender and maternal age, plus maternal age squared and place of origin. We also studied the predicted probabilities of MLPT and VPT given by the multivariate models in order to draw graphs which would help interpret the results. The likelihood ratio test was done to determine the goodness-of-fit of the models. Finally, since the total number of births was available, the prematurity prevalence was calculated. All analyses were performed at a 5% significance level, and associated confidence intervals (CI) were estimated for each relevant parameter. All the analyses were performed using IBM SPSS Statistics 19.0.

### Ethical questions

The neonatal screening program was implemented with informed parental consent. The use of these data was approved by the Ethics Committee of the Valencian Community. The data were supplied in an anonymized and encrypted file to maintain complete anonymity and confidentiality. Finally, the study was conducted according to the principles of the World Medical Association Declaration of Helsinki and complied with the norms in the European Union guidelines of good clinical practice.

## Results

The distribution of total newborn infants in the province of Alicante in the study period was as follows: 812, ≤ 32 weeks gestation; 4,483, 33-37 weeks; 69,997, ≥37 weeks. Of the 75,292 newborn infants, 5,295 were preterm according to the established criteria, a percentage of 7.0%, of which 1.1% were VPT and 6.0% were MLPT. The distribution of the study sample was: 588, ≤32 weeks gestation; 2,661, 33-37 weeks; and a control group of 1,075. However, the final sample excluded some infants due to lack of records for all the study variables: ≤32 weeks, 224; 33-37 weeks, 1,822; control, 125.

**Figure 1:**
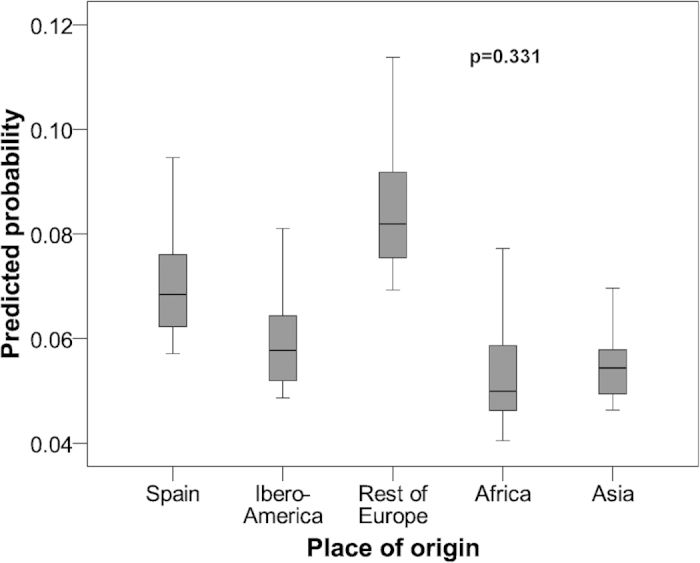
Predicted probability of being a moderate-to-late preterm infant in Alicante (Spain) according to mother’s place of origin. 2008-2011 data.

Analysis of the factors associated with a GA <37 weeks (MLPT) (Table 1) showed a significant association (p < 0.05) with maternal age (OR = 0.74, 95% CI: 0.63-0.88, p < 0.001) and its square (OR = 1.01, 95% CI: 1.00-1.01, p < 0.001). To clarify the association between place of origin and the parameter studied, the predicted probabilities of MLPT are shown in Fig. 1, which shows no differences according to the mother’s place of origin.

**Figure 2:**
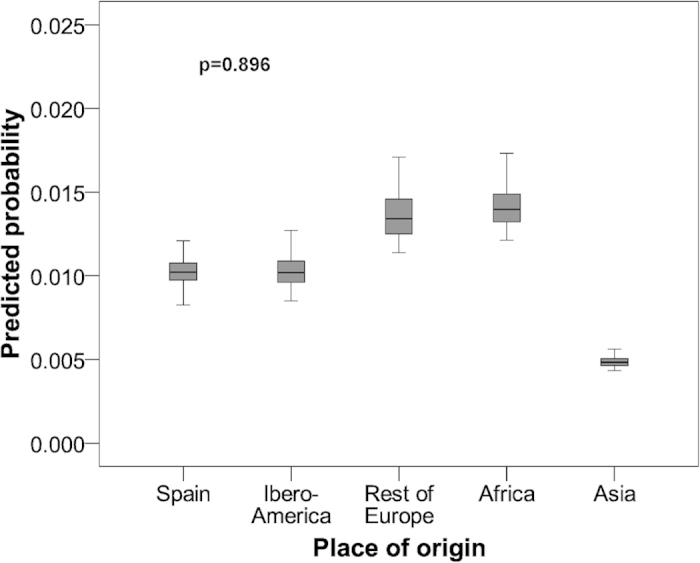
Predicted probability of being a very preterm infant in Alicante (Spain) according to mother’s place of origin. 2008-2011 data.

Analysis of the factors associated with VPT (Table 2) showed that no variables were significantly associated with the mother’s place of origin (p: 0.527-0.896). The predicted probabilities of VPT are shown in Fig. 2, which also shows that there were no differences according to the mother’s place of origin.

## Discussion

### Summary

This study showed that there was no statistically significant association between the mother´s origin and preterm births, either for MLPT (Fig. 1) or for VPT (Fig. 2).

### Strengths and limitations of the study

Of note was the statistical power, over 95%, when most studies use statistical powers between 80-90%. In addition, to minimize selection bias, the study used data from all preterm births and a random sample of full-term births. The information bias was minimized by collecting data precisely and carefully. Finally, no maternal variables were included that could affect prematurity, such as *in vitro* fertilisation, maternal disorders, or socio-economic factors^1^,^20^, as these data are not recorded for neonatal screening. Further studies may include these data.

### Comparison with the existing literature

We have compared our results with those found in studies undertaken in areas with similar healthcare systems, i.e., free and universal. Most of these are to be found in Europe and Canada^4^,^5^,^6^,^7^,^8^,^9^,^10^,^11^,^12^,^13^.

An interesting finding in the study by Urquia *et al.* was that recent immigrants had similar rates of prematurity as the indigenous population. However, the more years these immigrants had been in the country the greater the differences, with a higher proportion of prematurity among immigrants^4^,^5^. Considering that this type of immigration is a recent phenomenon in Spain^15^, our results are comparable to those reported in Canada^4^,^5^,^6^,^7^,^8^. They are also similar to the findings in Italy^10^, a country that, like Spain, has seen a recent increase in immigrants^21^. On the other hand, major immigration in France and the United Kingdom is not such a recent phenomenon as in Spain^21^, which may explain the differences in rates of prematurity between immigrants and natives^9^,^13^. Finally, studies undertaken in Portugal and Belgium only contemplated immigration from Africa, thus making direct comparison of the results difficult^11^,^12^.

Comparison of our results with those in an area with a completely different healthcare system, as is the United States, shows greater rates of prematurity among immigrants^14^.

### Implications for research and/or practice

This study shows the need to maintain the healthcare status of immigrants during the time they are in residence, by means of social integration measures that eliminate differences found in other countries, caused by socio-economic disparity^2^, where there are greater rates of prematurity the longer the immigrants remain in the country^4^,^5^,^6^,^7^,^8^.

## Conclusion

In a country with free universal healthcare cover, where the phenomenon of immigration is relatively recent, no differences were found in rates of prematurity depending on the mother’s origin. It is necessary to maintain this equality throughout the period of residence of immigrants by means of the implementation of social integration policies.

## Author Contributions

E.C. drafted the manuscript and A.P. performed the statistical analysis. E.C., M.M.R., A.P., M.J.A. and V.F.G. critically reviewed the manuscript and participated in the study design.

## Additional Information

**How to cite this article**: Cortés, E. *et al*. Influence of immigration on prematurity in the context of a free healthcare system with universal coverage. *Sci. Rep.* **5**, 10586; doi: 10.1038/srep10586 (2015).

## Supplementary Material

Supporting Information Supplementary Figures 1-6

## Figures and Tables

**Table 1:** Analysis of MLPT in the province of Alicante (Spain). 2008-2011 data.

**Variable**	**Total N** **=** **4,901**	**Cases n** **=** **3,837**	**Controls n** **=** **1,064**	**Adj. OR**	**95% CI Adj. OR**	**p-value**
Gender:						
Male	2,614(53.3)	2,077(54.1)	537(50.5)	1.18	(0.95,1.47)	0.140
Female	2,287(46.7)	1,760(45.9)	527(49.5)	1		

Place of origin:						
Ibero-America	372(7.6)	281(7.3)	91(8.6)	0.84	(0.55,1.29)	0.331
Rest of Europe	455(9.3)	374(9.7)	81(7.6)	1.23	(0.84,1.80)	
Africa	314(6.4)	229(6.0)	85(8.0)	0.70	(0.44,1.11)	
Asia	69(1.4)	51(1.3)	18(1.7)	0.80	(0.31,2.08)	
Spain	3,691(75.3)	2,902(75.6)	789(74.2)	1		
Mother’s age (years)	31.5 ± 5.6	31.5 ± 5.7	31.3 ± 5.1	0.74	(0.63,0.88)	<0.001
Mother’s age^2^ (years^2^)	1,201.1 ± 342.7	1,025.5 ± 351.0	1,005.3 ± 310.6	1.01	(1.00-1.01)	<0.001

Goodness-of-fit of the model: Χ^2^ = 18.8 p = 0.009.

Abbreviations: MLPT: moderate-to-late preterm infant; Adj. OR: Adjusted Odds Ratio; CI: confidence interval.

ORs were adjusted for: infant’s gender, mother’s age and place of origin.

**Table 2:** Analysis of very preterm infants in the province of Alicante (Spain). 2008-2011 data.

**Variable**	**Total N = 4,901**	**Cases n** = **588**	**Controls n** **=** **4,313**	**Adj. OR**	**95% CI Adj. OR**	**p-value**
Gender:						
Male	2,614(53.3)	325(55.3)	2,289(53.1)	N/M	N/M	N/M
Female	2,287(46.7)	263(44.7)	2,024(46.9)			

Place of origin:						
Ibero-America	372(7.6)	43(7.3)	329(7.6)	1.03	(0.36,2.95)	0.896
Rest of Europe	455(9.3)	67(11.4)	388(9.0)	1.36	(0.57,3.24)	
Africa	314(6.4)	48(8.2)	266(6.2)	1.44	(0.53,3.95)	
Asia	69(1.4)	4(0.7)	65(1.5)	0.50	(0.02,13.52)	
Spain	3,691(75.3)	426(72.4)	3,265(75.7)	1		
Mother’s age (years)	31.5 ± 5.6	31.7 ± 6.2	31.4 ± 5.5	N/M	N/M	N/M
Mother’s age^2^ (years^2^)	1,201.1 ± 342.7	1,042.1 ± 387.9	1,018.2 ± 336.1	1.00	(1.00-1.00)	0.527

Goodness-of-fit of the model: Χ^2^ = 1.4 p = 0.925.

Abbreviations: Adj. OR: adjusted odds ratio; CI: confidence interval; N/M, not in the model.

ORs were adjusted for: infant’s gender, mother’s age and place or origin.

To maintain the ratio between number of events and number of predictors ≥10, neither gender nor maternal age were included in the final model. The choice of variables in the model was based on the likelihood ratio test.
