# Supplementary material for: Influence of immigration on prematurity in the context of a free healthcare system with universal coverage
Source: Sci Rep. 2015 May 22;5:10586. doi: 10.1038/srep10586 (PMC4441151; doi:10.1038/srep10586)
Supplement: Supporting Information — Supplementary Figures 1-6 [file srep10586-s1.doc]

Title: Influence of immigration on prematurity in the context of a free healthcare system with universal coverage.

Running title: Immigration and prematurity in a Spanish region.

Authors: Ernesto Cortés^1^, María Mercedes Rizo-Baeza^2^, Antonio Palazón-Bru^3^, María José Aguilar^4^, Vicente Francisco Gil-Guillén^3^.

Countries included in the study:

Ibero- America: Colombia, Ecuador, Argentina, Paraguay, Bolivia, Uruguay, Venezuela, Brazil, Dominican Republic, Chile, Cuba, Mexico, Peru, El Salvador and Guatemala.

Rest of Europe: Romania, United Kingdom, France, Bulgaria, Russia, Germany, Ukraine, Netherlands, Poland, Italy, Belgium, Belarus, Lithuania, Switzerland, Portugal, Slovakia, Ireland, Croatia, Norway, Estonia, Moldova, Sweden, Georgia, Czech Republic and Serbia.

Africa: Morocco, Algeria, Nigeria, Senegal, Equatorial Guinea, Mauritania, Congo, Cape Verde, Cameroon, Ivory Coast, Egypt and Gambia.

Asia: China, India, Armenia, Pakistan, Philippines, Kazakhstan, Kyrgyzstan, Saudi Arabia, Azerbaijan, Iraq, Japan and Singapore.
